# A study on the potential of ants to act as vectors of foodborne pathogens

**DOI:** 10.3934/microbiol.2018.2.319

**Published:** 2018-04-20

**Authors:** Leckranee Simothy, Fawzi Mahomoodally, Hudaa Neetoo

**Affiliations:** Department of Agricultural and Food Sciences, Faculty of Agriculture, University of Mauritius, Réduit, Moka, 80837, Mauritius

**Keywords:** ants, food, pathogens, consumers, vectors

## Abstract

Ants (*Technomyrmex difficilis* and *Solenopsis geminata*) are insects often found in domestic kitchens of Mauritius. Unfortunately, they harbour disease-causing organisms and can potentially transfer these pathogens to food. This study was carried out to (i) investigate the knowledge, perception and behaviors of consumers in relation to the problem of ant infestation of domestic kitchens; (ii) identify the pathogenic microorganisms carried by ants; and (iii) determine the potential for ants to transfer these pathogenic microorganisms to food. A survey based on a stratified sampling design was carried out with 100 consumers using a questionnaire. To identify the pathogenic microorganism(s) harbored by ants, bait traps were set up using sterile sugar as a non-toxic attractant. Captured ants were then subjected to microbiological analyses. Most respondents (72%) agreed that ants constitute a hygienic issue but they did not perceive ants as a serious threat to human health. However microbiological analyses of ants (n = 50) confirmed the presence of various pathogenic microorganisms as well as fecal contaminants. Ants were found to harbor yeasts and molds systematically (100%), coliforms frequently (52%), *Bacillus* spp. and *Escherichia coli* occasionally (26% and 18% respectively) and *Salmonella* and *Listeria monocytogenes* sporadically (8 and 6 % respectively). Ants were also found to transfer *E. coli* to food surfaces at a moderately high frequency of 70%. This study demonstrated that the majority of consumers acknowledged the problem of ant infestation as a sanitation-related problem rather than a food safety issue. Since ants have the ability to harbor and subsequently transfer pathogenic or toxigenic microorganisms, ants may act as disease vectors and contaminate food, water and food- contact surfaces of kitchens resulting in foodborne illnesses.

## 1. Introduction

Ants (*Formicidae*, Hymenoptera) are ubiquitous social insects that live in colonies. Female worker ants typically leave the nest and venture out in search for food [1]. Ants are generally omnivorous and the animal proteins and fats in their diet are derived mostly from insects and other arthropods that fall prey to the foraging worker ants [1]. Ants also feed on sugars, starches or foods containing those carbohydrates. As a result, kitchens, bakeries, restaurants, and food factories are typical sites for foraging ants [2]. Although ants are significant vectors of infectious diseases, there is considerably less information on ants than on other insect pests such as flies and cockroaches. Unfortunately, ants harbor various species of internal [3],[4] as well as external [1],[5],[6] bacteria, which adhere to the external surfaces, mainly the legs and mandibles [1],[5],[6]. These appendages come into contact with substrates, such as soil and pit latrines outdoors and, most commonly, floors indoors, from which the ants may pick up pathogens [1]. As ants forage over clean food-contact surfaces, such as dishes and cutting boards, pathogens may be deposited and eventually become mixed in with a ready-to-eat food intended for human or animal consumption. Literature has indicated that multiple pathogens have been isolated from pest ants including *Bacillus cereus* [7],[8], *Clostridium perfringens* [7], *Escherichia coli* [7], filamentous fungi [8], *Klebsiella pneumoniae* [9], *Micrococcus* sp. [8], *Proteus mirabilis* [9], *Pseudomonas* spp. [9], *Salmonella* sp. [7], *Staphylococcus aureus* [7], other *Staphylococcus* spp. [8] and *Streptococcus pyogenes* [7]. Unfortunately, ants have the potential to carry certain microorganisms to food establishments and transfer pathogenic microorganisms to food [6].

According to WHO [10], food safety is an essential aspect in public health and foodborne disease outbreaks and epidemics have been classified as a major global public health issue in the 21^st^ century. As ants are major mechanical vectors of pathogens [11], including foodborne pathogens [8], it is important to exterminate them in kitchens and other food preparation facilities so as to prevent contamination of food. The problem of ant infestation can be addressed by preventing their ingress into homes [12]. This can be achieved by filling or sealing crevices and cracks, cleaning around common entry points with a detergent to remove the chemical trail of pheromone along their routes to and from a food source, or using a non-repellent residual insecticide [13]. Previous studies have demonstrated that proper knowledge, attitude and practices among consumers are the key elements to ensure food safety [12]. However, there is currently a dearth of information on consumers' knowledge, perception and behavior in relation to pest ants in domestic kitchens as little research has been carried out on this topic. The purpose of this study was therefore to (i) provide insight on consumers' knowledge, perception and behaviors regarding infestation of domestic kitchen by ants; (ii) identify pathogenic microorganisms that are harbored by ants which regularly infest kitchens; and (iii) determine the potential of insects to transfer microorganisms to food.

## 2. Materials and methods

### 2.1. Consumer survey

A survey questionnaire was designed to shed light on consumers' knowledge, perception and behavior regarding pest ants in domestic kitchens. In this investigation, 100 participants were selected randomly to represent Mauritian consumers. The target population for this survey was members of the general public of both genders, from diverse educational and ethnic backgrounds. The participants were contacted personally at their place or workplace and requested to fill in the questionnaire appropriately with answers of their choice. They were also informed that all the information collected during the course of this survey would be kept confidential and strictly used for statistical purpose. The questionnaire contained items pertaining to demographic information such as age, gender and marital status as well as a series of close-ended and open-ended questions assessing the knowledge, perception, behavior and control practices of pest ants in domestic kitchens.

### 2.2. Determination of microbial carriage of ants

Microbiological tests were carried out in order to identify any pathogenic microorganisms found on ants commonly present in domestic kitchens. The method of capturing and microbiologically analyzing ants was adapted from Maximo et al. [14] and Ogba et al. [15] respectively. Briefly, 50-ml centrifuge tubes, each containing 10 g of sugar, were heat-sterilized to act as baits. Ants were collected during the period of September–October 2016 from 5 different domestic kitchens located in the Northern district of Pamplemousses. This region was chosen since the climate is warmer and relatively more humid, rendering it more conducive for ant breeding and house infestation. The tubes were left on kitchen countertops near (i) windowsills, (ii) sinks, (iii) bread-storage areas, (iv) pantries or (v) microwaves overnight for ant collection. These areas were selected because of the higher incidence of ants as reported by survey-participants and the relative ease of capturing them. Two independent trials were carried out totaling 50 samples (5 kitchens × 5 sampling sites × 2 replicates). Once ants were captured, tubes were capped and refrigerated at 4 °C for 1 h to immobilize the insects. These ants were identified taxonomically as *Technomyrmex difficilis* and *Solenopsis geminata* by consulting the resource AntWeb v. 7.7.4 (antweb.org) as well as comparison of their key features with a collection of ant specimens from the Zoology Laboratory of the University of Mauritius. Their identity was further confirmed by consulting a local entomologist.

For the microbiological analysis of ants, 40 ml of 1.0% sterilized buffered peptone water (Hi-Media) was added to each tube and incubated for 24 h at 35 °C for enrichment. A loopful of the enriched culture was streaked on Violet Red Bile Agar, Eosin Methylene Blue Agar, Slanetz and Bartley Medium, Bacillus Agar, Iron sulphite Agar, McBride Listeria Agar, Xylose Lysine Desoxycholate Agar and Potato Dextrose Agar (PDA) to detect the presence of coliforms, *Escherichia coli*, *Enterococcus faecalis*, *Bacillus* species, *Clostridium perfringens*, *Listeria monocytogenes*, *Salmonella* spp. and yeasts and molds respectively. Plates were incubated at 35 °C for 24 h after which they were observed for presence or absence of growth.

### 2.3. Determination of transference rate of tracer bacteria E. coli to food by ants

**Figure 1.**
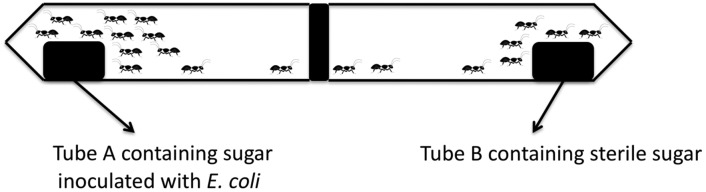
Interconnected chamber systems for studying microbial transference by captured ants.

This experiment was carried out to determine whether ants have the potential to transfer pathogenic bacteria from a contaminated source to food. Twenty 50-ml centrifuge tubes were autoclaved, to which sterile sugar (10 g) was subsequently added to serve as an attractant. Sugar from ten of the tubes was inoculated with 200 µL of a late-log phase culture of *E. coli* ATCC 25922 to a final population density of ca. 10^7^ cfu/g. These tubes were referred to as “Tubes A”. These tubes were then left open overnight on countertops of five different kitchens (2 tubes per kitchen) to collect ants. After collection, the ten tubes containing ants were quickly juxtaposed with tubes containing only sterile sugar (Tubes B) according to the set-up shown below (Figure 1). The adjacent mouths of the tubes were then taped together using scotch tape.

The set-up was left on kitchen countertops for 24 h at ambient temperature to allow enough time for ants from the set of Tubes A to migrate to adjacent Tubes B. After 24 h, the tape fixing the pairs of tubes was subsequently removed and captured ants were set free. The sugar content of all 20 tubes was then microbiological analyzed by enrichment in 40 ml of 1.0% sterilized buffered peptone water, followed by incubation at 35 °C for 24 h. A loopful of the enriched samples was then streaked on EMB agar to determine presence of *E. coli*.

### 2.4. Statistical analysis

Survey data were analyzed by descriptive statistics and Pearson's bivariate correlation using SPSS version 16.0. Figures and tables were drawn using Microsoft Excel 2010. For the microbiological tests, results were recorded as presence/absence and tabulated using Microsoft Excel 2010. For statistical analysis of microbial carriage data, presence and absence of the different genera were transformed into codes of 1 and 0 respectively before being subjected to a two-way ANOVA using GraphPad Prism 7 to assess differences and interactions due to the “kitchen effect” and “sampling site effect”. Differences in the mean prevalence among kitchens or sampling sites were calculated using the Tukey Multiple Comparisons test at the alpha level of 0.05. To determine if the overall prevalence of the different microbial groups was statistically significant, coded data were analyzed using Mann-Whitney U test (GraphPad Prism 5). For statistical analysis of transference data, presence/absence of tracer organism *E. coli* in the 10 pairs of Tube A and B were transformed as described previously and subjected to a Wilcoxon Matched-Pairs Signed Rank Test. Transference rates were calculated using the formula:

$$\text{Transference rate }\left(\text{\%}\right)=\frac{\text{Number of positive B tubes}}{\text{Number of positive A tubes}}\times 100$$ (1)

## 3. Results and discussion

### 3.1. Survey results

In this study, the majority of survey participants had a good educational background with either a certificate (37%), undergraduate degree (24%) or diploma (19%), with a minority with either a school certificate or a postgraduate degree. The 88% of participants correctly responded that ant infestation means presence of large numbers of ants in multiple places in the house while 7% thought that it refers to the mere presence of a few ants and the rest (5%) of the participants did not know the answer. In fact, we observed a significant (*p* < 0.05) but very weak positive relationship (Pearson's R = 0.139) between “level of education” and “problems associated with ant infestation”. This finding reveals that participants with a higher level of education were slightly more concerned about the general unaesthetic issues associated with ant infestation. However, there was no significant (*p* > 0.05) correlation between “level of education” of participants and their appreciation of the “potential of ants to act as disease vectors” and “potential of ants to transmit germs to food” or “risks of microbial cross-contamination of food surfaces by ants”.

Consumers perceived different pests with varying degrees of concern in the following decreasing order: rats (71%) > lizards (13%) > ants (9%). Indeed, the three main groups of pests often encountered in kitchens are rodents (rats and mice), followed by insects (cockroaches, beetles, ants and flies) and birds with pigeons being the main bird pest [16]. The relatively little concern expressed by survey participants with regard to ants is congruent with observations made by Monteiro de Castro [17] who noted that human behavior varied from either extreme entomophobia or total disregard towards ants.

Table 1 shows that most participants (72%) felt that ants represented a nuisance and 50% of them perceived indoor ants of kitchens to be a greater source of disturbance than their outdoor counterparts. However, the majority of respondents (86%) had no allergy, intolerance or idiosyncrasy towards ants. Many of them (62%) also did not consider ants as a serious threat to public health. In fact, there was no statistically significant (*p* > 0.05) correlation (Pearson's R of −0.005) between consumers' perceived “problems associated with ant infestation” and the belief that “ants are a threat to public health”. In other words, most consumers who regarded ant infestation of domestic kitchens to be a source of disturbance did not necessarily relate it to health-related issues. In fact, more emphasis has been laid in the literature on the high economical importance of ants because of damages caused to crops, buildings and electrical appliances [17],[18],[19]. Indeed, the economic cost of fire ants in the United States is an estimated US$ 6.5 billion annually, with the majority of the losses in the urban sector [20]. However, there was a statistically significant (*p* < 0.05) and positive medium-strength association (Pearson's R of 0.412) between the beliefs that “ants are a threat to public health” and “ants are reservoirs of germs”. In other words, most respondents who acknowledged ants as compromising public health (38% of participants) also appreciated their role in carrying disease-causing microorganisms.

**Table 1.** Perception of consumers on ants.

Concern about ants	Responses	Proportion (%)
Ants are a source of nuisance	Yes	72
	No	28
Ants inside kitchens are a greater source of disturbance than outdoor ants	Yes	50
	No	50
Participant has allergy, intolerance or idiosyncrasy	Yes	14
	No	86
Ants is a serious threat to public health	Yes	38
	No	62

**Figure 2.**
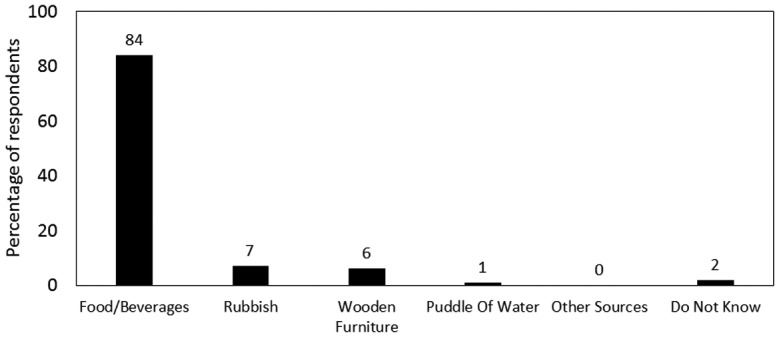
Perceived factors contributing to ant infestation.

Figure 2 below shows the relative contribution of different factors perceived by consumers to be responsible for ant infestation in the kitchen. The majority of the sample population (84%) thought that the main factors that contribute to ant infestation in the kitchen are food and beverages while only few participants believed that rubbish (7%) and wooden furniture (6%) were the main sources respectively. This might imply that ants found in Mauritius are mainly attracted to foodstuffs found in kitchens. Indeed, Gorham also mentioned that foraging ants are primarily attracted to foods especially those rich in carbohydrates [21]. Kitchen is thus a breeding place for ants as most foodstuffs are stored there. Thus, cross contamination might also be occurring as ants come in contact with exposed food-contact surfaces. Fifty percent of participants indicated “sight of ants on food and beverages” to be the main reason for control of ants, while others pointed to the fact that “ants transmit germs” (22%), “ants are a source of embarrassment in front of guests” (16%) or “ants bite or sting” (12%) as the main drivers. Hence consumers appeared to be far more concerned by the compromised aesthetic appeal of the food rather than its microbiological safety. This is in contradiction with findings of Cicatiello et al. [22] who mentioned that the majority of subjects (82.5%) believed that insects were main sources of bacteria being brought into the kitchen.

As far as breeding season was concerned, participants thought that ants had a tendency to breed during summer (68%), during both summer and winter (20%) or during the winter season (3%). The remaining participants indicated not knowing during which season ants tend to breed. In fact, *Technomyrmex albipes* tend to reproduce more rapidly in warm weather and immature ants also develop faster at warmer temperatures [23]. Lutinski et al. [24] also indicated that warmer temperatures are more conducive for breeding and infestation of ants. In fact, seasonality not only affects ant abundance but also ant diversity in hospital settings [25].

**Figure 3.**
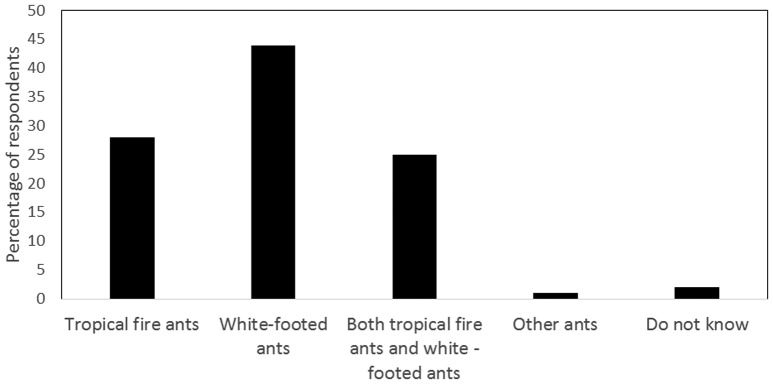
Types of ants found in domestic kitchens.

Figure 3 shows that 44% of respondents mentioned that white-footed ants (commonly referred to as “black ants”) (*Technomyrmex difficilis*), were the predominant type found in their kitchen. Previously identified as Technomyrmex albipes (Fr. Smith), it was correctly identified in 2007 as Technomyrmex difficilis [26]. The 28% of survey-takers indicated tropical fire ants (*Solenopsis geminata*) to be the dominant specie while 25% of survey-participants mentioned that both species could be found in their kitchens. Only a minority (1%) found other types of ants in kitchen. In fact, both white-footed ants and fire ants are considered as tramp species with synanthropic behavior, thus allowing them to successfully disperse in urban areas and live in close association with humans [27],[28].

**Figure 4.**
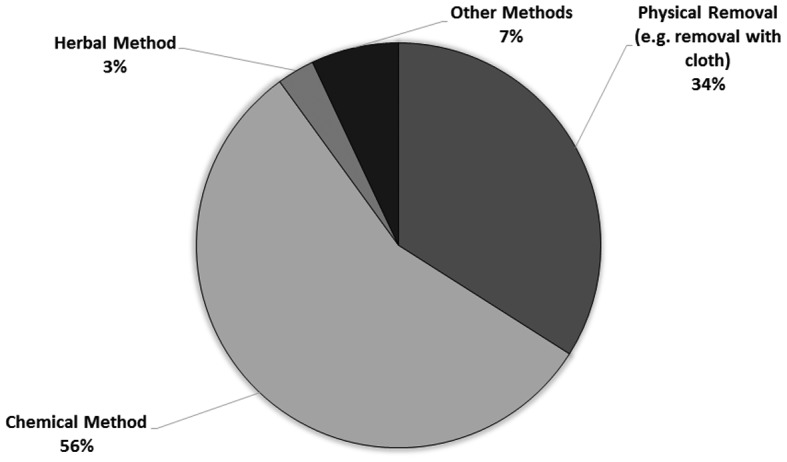
Ant control methods.

The pie chart (Figure 4) below shows the different methods adopted by consumers to control ant infestation. The 56% of consumers made use of chemicals to ward off ants while 34% stated that they preferred to physically remove them. A minority of participants responded using other methods (7%) including herbal methods (3%) to get rid of ants. In fact, Monteiro de Castro [17] mentioned that most conventional treatments only have temporary effects, because they eliminate only part of the colony. Several researchers have indicated that an efficient control program should be based on the complete elimination of the colony. According to Bueno and Fowler [29] toxic baits stand out as the most efficient treatment because the insecticide is incorporated in the feeding cycle of the colony. Nickerson et al. [30] on the other hand pointed to a more preventive approach for controlling ants at home by ensuring tidiness and cleanliness of the house, since unnecessary clutter can create harborage sites for hidden nests.

### 3.2. Microbial carriage of ants

**Figure 5.**
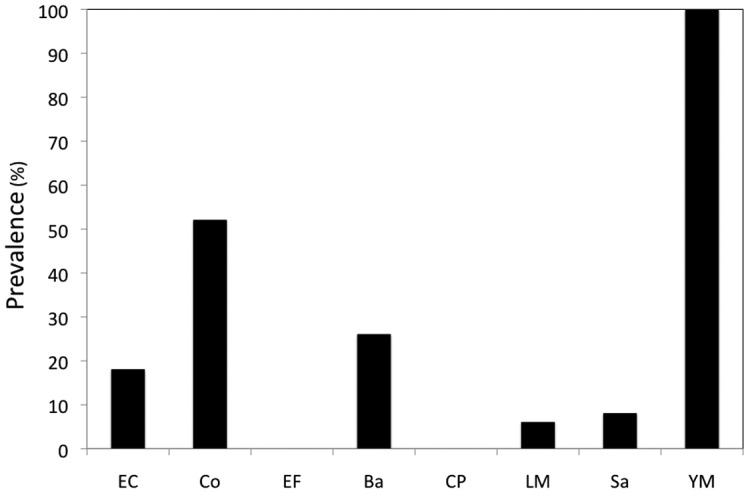
Frequency of occurrence of different microorganisms on ants. EC: *E. coli*; Co: Total coliforms; EF: *E. faecalis*; Ba: *Bacillus* spp.; CP: *C. perfringens*; LM: *L. monocytogenes*; Sa: *Salmonella* spp.; YM: Yeasts and molds.

With regards to microbial diversity of ants, yeasts and molds were consistently present (100%) in all samples followed by coliforms (52%), *Bacillus* spp. (26%), *E. coli* (18%), presumptive *Salmonella* spp. (8%) and *L. monocytogenes* (6%) (Figure 5). However, *E. faecalis* and *C. perfringens* were undetected throughout (Figure 5). Statistical analysis indicated that ants collected from domestic kitchens were significant carriers of Yeasts and Molds, coliforms and *Bacillus* spp. (*p* < 0.05) while occurrence of *E. coli*, *Salmonella* spp. and *L. monocytogenes* on ants was not significant (*p* > 0.05).

Table 2 through Table 9 show the prevalence of different microbial groups collected on ants sampled from different sites and kitchens.

**Table 2.** Prevalence of *E. coli* from ants collected in various sites and kitchens.

Site	Kitchen				
	A	B	C	D	E
Window sill	(1/2)	(0/2)	(0/2)	(0/2)	(0/2)
Sink	(1/2)	(0/2)	(1/2)	(1/2)	(1/2)
Bread storage	(0/2)	(0/2)	(0/2)	(1/2)	(1/2)
Pantry	(1/2)	(0/2)	(1/2)	(0/2)	(0/2)
Microwave	(0/2)	(0/2)	(0/2)	(0/2)	(0/2)

Numbers in brackets represent the number of samples testing positive for *E. coli* out of two.

**Table 3.** Prevalence of *E. faecalis* from ants collected in various sites and kitchens.

Site	Kitchen				
	A	B	C	D	E
Window sill	(0/2)	(0/2)	(0/2)	(0/2)	(0/2)
Sink	(0/2)	(0/2)	(0/2)	(0/2)	(0/2)
Bread storage	(0/2)	(0/2)	(0/2)	(0/2)	(0/2)
Pantry	(0/2)	(0/2)	(0/2)	(0/2)	(0/2)
Microwave	(0/2)	(0/2)	(0/2)	(0/2)	(0/2)

Numbers in brackets represent the number of samples testing positive for *E. faecalis* out of two.

**Table 4.** Prevalence of coliforms from ants collected in various sites and kitchens.

Site	Kitchen				
	A	B	C	D	E
Window sill	(0/2)	(1/2)	(1/2)	(0/2)	(0/2)
Sink	(1/2)	(2/2)	(1/2)	(2/2)	(1/2)
Bread storage^†^	(2/2)	(2/2)	(2/2)	(1/2)	(2/2)
Pantry	(1/2)	(1/2)	(1/2)	(1/2)	(0/2)
Microwave	(0/2)	(1/2)	(2/2)	(1/2)	(0/2)

Numbers in brackets represent the number of samples testing positive for coliforms out of two. ^†^: Superscript symbol indicates site with statistically higher mean prevalence of coliforms.

**Table 5.** Prevalence of *Bacillus* spp. from ants collected in various sites and kitchens.

Site	Kitchen				
	A	B	C	D	E
Window sill	(0/2)	(0/2)	(0/2)	(0/2)	(0/2)
Sink^†^	(0/2)	(1/2)	(1/2)	(2/2)	(2/2)
Bread storage	(1/2)	(1/2)	(0/2)	(0/2)	(0/2)
Pantry	(0/2)	(0/2)	(1/2)	(0/2)	(0/2)
Microwave	(0/2)	(1/2)	(2/2)	(1/2)	(0/2)

Numbers in brackets represent the number of samples testing positive for *Bacillus* spp. out of two. ^†^: Superscript symbol indicates site with statistically higher mean prevalence of *Bacillus* spp.

**Table 6.** Prevalence of *Salmonella* spp. from ants collected in various sites and kitchens.

Site	Kitchen				
	A	B	C	D	E
Window sill	(0/2)	(0/2)	(1/2)	(0/2)	(0/2)
Sink	(0/2)	(1/2)	(1/2)	(1/2)	(0/2)
Bread storage	(0/2)	(0/2)	(0/2)	(0/2)	(0/2)
Pantry	(0/2)	(0/2)	(0/2)	(0/2)	(0/2)
Microwave	(0/2)	(0/2)	(0/2)	(0/2)	(0/2)

Numbers in brackets represent the number of samples testing positive for *Salmonella* out of two.

**Table 7.** Prevalence of Yeasts and Molds from ants collected in various sites and kitchens.

Site	Kitchen				
	A	B	C	D	E
Window sill	(2/2)	(2/2)	(2/2)	(2/2)	(2/2)
Sink	(2/2)	(2/2)	(2/2)	(2/2)	(2/2)
Bread storage	(2/2)	(2/2)	(2/2)	(2/2)	(2/2)
Pantry	(2/2)	(2/2)	(2/2)	(2/2)	(2/2)
Microwave	(2/2)	(2/2)	(2/2)	(2/2)	(2/2)

Numbers in brackets represent the number of samples testing positive for yeasts and molds out of two.

**Table 8.** Prevalence of *L. monocytogenes* from ants collected in various sites and kitchens.

Site	Kitchen				
	A	B	C	D	E
Window sill	(0/2)	(0/2)	(0/2)	(0/2)	(0/2)
Sink	(0/2)	(0/2)	(1/2)	(0/2)	(0/2)
Bread storage	(0/2)	(1/2)	(0/2)	(1/2)	(0/2)
Pantry	(0/2)	(0/2)	(0/2)	(0/2)	(0/2)
Microwave	(0/2)	(0/2)	(0/2)	(0/2)	(0/2)

Numbers in brackets represent the number of samples testing positive for *Listeria monocytogenes* out of two.

**Table 9.** Prevalence of *C. perfringens* from ants collected in various sites and kitchens.

Site	Kitchen				
	A	B	C	D	E
Window sill	(0/2)	(0/2)	(0/2)	(0/2)	(0/2)
Sink	(0/2)	(0/2)	(0/2)	(0/2)	(0/2)
Bread storage	(0/2)	(0/2)	(0/2)	(0/2)	(0/2)
Pantry	(0/2)	(0/2)	(0/2)	(0/2)	(0/2)
Microwave	(0/2)	(0/2)	(0/2)	(0/2)	(0/2)

Numbers in brackets represent the number of samples testing positive for *C. perfringens* out of two.

Kitchens and sampling sites within each kitchen did not have any differential effect on ants' carriage of *E. coli*, *E. faecalis*, *C. perfringens*, Yeasts and Molds, *Salmonella* and *L. monocytogenes* of ants since the “kitchen”, “sampling area” and “kitchen* sampling area interaction” effects were not significant (*p* > 0.05). However, for *Bacillus* spp. and coliforms, the “sampling site” was found to be a significant factor (*p* < 0.05). Tukey's Multiple Comparisons tests revealed that ants collected near bread storage areas and sinks were significantly more frequent carriers of (*p* < 0.05) of coliforms and *Bacillus* spp. respectively. Since the microflora of ants could very well reflect the microbial ecology of the kitchen areas themselves, this finding points to potential harborage areas for certain microbial species.

The systematic presence of fungi on ants noted in the current investigation is in agreement with a study conducted by De Zarzuela et al. [6] in residential kitchens and bathrooms whereby out of 137 ants, 66 (48.2%) were contaminated with fungi. Monteiro de Castro [17] also conducted a meta-analysis of studies assessing the association between ants and microorganisms and determined that 38% of investigations reported association with fungi. Tanada and Kaya [31]–[34] further reported presence of toxigenic molds such as *Aspergillus* spp. on edible insects. Even the potential for transmission of fungi by ants has been demonstrated [31],[32].

In addition to fungi, our findings reveal fire ants and white-footed ants as vectors of bacteria including coliforms, *Bacillus*, *E. coli*, *Salmonella* and *L. monocytogenes*. In fact, 72% of studies on the microbiota of ants reported association of ants with bacterial pathogens [17]. Other studies similarly identified fecal coliforms such as *Enterobacter*, *Escherichia* and *Klebsiella* spp., as well as pathogenic bacteria *S. aureus* on ants [32],[33]. Ogba et al. [15] isolated a total of 205 species from Banded sugar ants (*Camponotus consobrinus*) and determined *E. coli* (fecal indicator bacteria; 30%) to be the most common isolate, followed by *Morganella morganii* (histamine-forming bacteria, 18%), *Serratia marcescens* (opportunistic pathogen, 4%) and *Citrobacter freundii* (coliform, 4%). Rodovalho et al. [18] also isolated gram-negative bacilli at a carriage rate of 16.7% from ants (*Tapinoma melanocephalum* and *Camponotus vittatus*) and concluded that these insects may be responsible for carrying as well as distributing gram-negative bacilli in the hospital environment. In fact, ants can get into contact with infectious human materials including feces, urine and sputum [18], and subsequently transfer these bacterial cells to kitchens' utensils and surfaces [32]. Smith [12] on the other hand tested tropical fire ants (*Solenopsis geminata*) for the presence of dysentery bacteria and demonstrated the presence of viable *Shigella* spp. Tanada and Kaya [34] thus reported that the insect microbiome is very diverse and complex; some microorganisms acting as vital symbionts and others as powerful entomo-pathogens. It is likely that microorganisms isolated from ants, whether pathological or physiological, could be indigenous to the insects (autochthonous) or acquired from the environment (allochthonous).

### 3.3. Microbial transference potential of ants

The transference experiment revealed that 7 out of 10 originally sterile sugar samples became contaminated with *E. coli* after exposure to ants; in other words the transference rate was 70%. This points to a statistically significant (*p* < 0.05) vector potential of ants in disseminating microorganisms from contaminated to uncontaminated matrices. *E. coli* is known to survive in water and certain ants such as white-footed ants have an affinity for water [35]. This explains their abundance in houses where they search in long trails for water [35]. Since kitchens contain damp areas such as sinks, wet sponges, table surfaces or floors, these can be ideal harborage sites for both white-footed ants and diarrheagenic *E. coli*. As a result, ants can easily move through these places and contaminate food in kitchens.

Findings on the phenomenon of ant-mediated bacterial transference reported here in fact corroborate observations made by Fowler et al. [36] in his pioneering work on the transmission potential of pathogens by ants in hospital settings. Similar to our study, Wasala et al. [37] showed that house-flies (*Musca domestica*) were capable of transferring *E. coli* O157:H7 to spinach. Another study revealed that flies inoculated with green fluorescent protein (GFP)-tagged *E. coli* were capable of transmitting *E. coli* to intact apples in a cage model system [38]. These data support the hypothesis that arthropods are potential vectors of pathogenic microorganisms onto food. Gazeta et al. [39] further specified that arthropods such as ants, cockroaches and flies are main vectors of infectious microorganisms by virtue of their contact with human feces and other contaminated materials. Since sewage and landfills are major reservoirs of pathogenic microorganisms, lack of investment in basic sanitation and pest control can therefore lead to serious public health problems [40]. Along the same line, Beatson [7] also identified ants as potential vectors of pathogens and thus inferred that ant infestation in homes and hospitals is a risk to public health.

Taken together, this study highlights the significance of ants as systematic carriers of fungi, frequent carriers of coliforms and *Bacillus spp*., and sporadic carriers of *Salmonella* and *L. monocytogenes*. Moreover, this study demonstrated that ants have the potential to transfer pathogenic agents from contaminated matrices to food. This issue is further compounded by the general behavior of Mauritians towards ants, which varied from mild concern to total disregard leading the society to believe that there is no need for ant monitoring and control. In fact, most consumers approached in the study were not aware of any health implications associated with ant infestation of kitchens and exposed food, making them more vulnerable to foodborne illnesses. Since ants are prevalent in homes, particularly in food preparation and storage areas, concern about their impact on food quality and safety is certainly warranted.

A few limitations of the study should however be noted. Firstly, unlike other researchers, we did not perform a visual assessment of the kitchens' level of cleanliness and hygiene or an environmental monitoring of kitchen surfaces by swabbing. This could have shed more light on the role of ants in the epidemiology of foodborne pathogens. Moreover, the sample size for ant sampling and analyses were relatively small compared with most published studies in this area. Thirdly, unlike several studies, which reported the microbial population density, our approach was more qualitative as we reported the presence/absence of specific microbial genera. Fourth, we did not analyze ants for coagulase-positive staphylococci unlike other researchers and this additional microbiological parameter could have provided insight on the possible role of ants to cause contamination of food and eventual toxin-production by *S. aureus*. Finally, antibiotic-susceptibility testing, molecular identification of these isolates and detection of mycotoxins could have been performed. Nevertheless, the current study is the first to be carried out in Mauritius that demonstrates the vector potential of fire ants and white-footed ants in household settings. Findings reported in this work can pave the way for future research and development, testing and validation of more efficient ant control programs. Indeed, current methods are too weak and only lead to population booms by reducing diversity and competition among species [41]. Additionally, education and sensitization campaigns of communities on adoption of proper sanitation and pest control methods are highly recommended.

## 4. Conclusions

The aim of this study was to investigate the knowledge, perceptions and behaviors of consumers towards infestation of domestic kitchens by ants and to shed light on the microbial diversity of these insects. Findings of this study indicated that the majority of consumers were cognizant of various problems posed by ant infestation. However, consumers did not generally perceive ants as carriers of pathogenic microorganisms, nor as presenting a risk to public health. Microbiological analyses of ants captured from domestic kitchens revealed the presence of various microbial groups including coliforms, *Bacillus* spp., *E. coli*, *Salmonella* spp., *Listeria* spp. and fungi. This study further highlighted the potential for ants to transmit pathogenic microorganisms from contaminated environments to food. Mauritian consumers should thus be increasingly sensitized on the role of ants in the epidemiology of foodborne diseases as well as the use of effective methods to combat ant infestation.
