# Supplementary material for: A study on the potential of ants to act as vectors of foodborne pathogens
Source: AIMS Microbiol. 2018 Apr 20;4(2):319–33. doi: 10.3934/microbiol.2018.2.319 (PMC6604928; doi:10.3934/microbiol.2018.2.319)
Supplement: Supplementary file 1 [file microbiol-04-02-319-s1.pdf]

AIMS Microbiology, 4(2): 319–333.
DOI: 10.3934/microbiol.2018.2.319
Received: 24 January 2018
Accepted: 02 April 2018
Published: 20 April 2018

http://www.aimspress.com/journal/microbiology

Research article

A study on the potential of ants to act as vectors of foodborne pathogens

Leckranee Simothy, Fawzi Mahomoodally and Hudaa Neetoo*

Department of Agricultural and Food Sciences, Faculty of Agriculture, University of Mauritius, Réduit, Moka, 80837, Mauritius

* **Correspondence:** Email: s.neetoo@uom.ac.mu; Tel: +2304037885; Fax: +2304655743.

Supplementary

The purpose of this survey is to assess the knowledge, perception and behavior of consumers regarding pest ants found in kitchens. Please be informed that all information concerning the participants will be kept strictly confidential.

Please respond to the questions below as accurately as you can

1. (i) Gender (ii) Age: ……….

   Male ☐ Female ☐

2. Marital status:

   Single ☐ Divorced ☐

   Married ☐ Others ☐

3. Highest qualification: …………………………

4. Do you work?

   Yes ☐ No ☐

5. What is ant infestation? (Tick one answer only)

   ☐ Presence of group of ants in multiple places in the house

   ☐ Presence of one or two ants in the house

   ☐ Do not know

6. Which problem is associated with ant infestation?

   ☐ A. These cause nuisances to humans (e.g damaging wooden furniture)

   ☐ B. These transmit diseases to human

   ☐ C. Do not know

7. If you have checked answer B above, what is the mode of disease transmission?

   ☐ Contaminate food

   ☐ Cause infection through wounds

   ☐ Do not know

8. According to you in what season do ants breed more?

   ☐ Summer

   ☐ Winter

   ☐ Do not know

9. Which type of ant is mostly found in the kitchen?

   ☐ Fire ants (“fourmi rouge”)

   ☐ White-footed ants (“fourmi noir”)

   ☐ Both fire and white-footed ants

   ☐ Other ants

   ☐ Do not know

10. Where in the kitchen are ants mostly found?

   ☐ Near window sills

   ☐ Near sink

☐ Near bread-storage area

☐ Near pantry (food storage area)

☐ Near stove

☐ Other

11. According to you, which factor most contributes to ant infestation in kitchens?

☐ Food/beverages

☐ Rubbish

☐ Wooden furniture

☐ Puddle of water

☐ Other sources

☐ Do not know

12. Does the presence of ants bother you?

☐ Yes ☐ No

13. Do you see ants as a serious nuisance?

☐ Yes ☐ No

14. Do you think that ants are a threat to public health?

☐ Yes ☐ No

15. Do you have any allergy, intolerance or idiosyncrasy towards ants?

☐ Yes ☐ No

16. Do you know a disease or infection that is transmitted by ants?

☐ Yes ☐ No

17. How do you get rid of ants?

☐ Physical removal (e.g removal with cloth)

☐ Use of chemical methods (e.g use of insecticide)

- ☐ Use of herbal methods (e.g use of cloves)
- ☐ Other methods

18. According to you what is the best method to control ants?

- ☐ Proper sanitation
- ☐ Ant bait
- ☐ Other method
- ☐ Do not know

19. What is the main reason for ant control?

- ☐ Embarrassment in front of guests
- ☐ Ants transmits germs
- ☐ Ant in food/beverages
- ☐ Biting/stinging

20. Do you think that ants are a reservoir of disease-causing germs?

☐ Yes ☐ No

21. Do you think that ants are a vector of disease-causing germs?

☐ Yes ☐ No

22. Do you think ants can transmit germs to food?

☐ Yes ☐ No

23. How can you prevent food from ant cross contamination?

- ☐ Storing food in refrigerator
- ☐ Covering food properly
- ☐ Proper sanitation of kitchen tools and surfaces
- ☐ Do not know

24. What is most important pest problem according to you?

- ☐ Ant problem
- ☐ Rat problem
- ☐ Lizard problem
- ☐ Others

25. Can ants be eliminated?

☐ Yes ☐ No

Additional Comments:

AIMS Press

© 2018 the Author(s), licensee AIMS Press. This is an open access article distributed under the terms of the Creative Commons Attribution License (http://creativecommons.org/licenses/by/4.0)
